# 25 vs. 27-gauge micro-incision vitrectomy surgery for visually significant macular membranes and full-thickness macular holes: a retrospective study

**DOI:** 10.1186/s40942-020-00259-4

**Published:** 2020-11-16

**Authors:** Gordon T. Brown, Sangeethabalasri Pugazhendhi, Robert M. Beardsley, John W. Karth, Peter A. Karth, Allan A. Hunter

**Affiliations:** Oregon Eye Consultants LLC, 1550 Oak St, Suite 7, Eugene, OR 97401 USA

**Keywords:** 25-gauge, 27-gauge, Epiretinal membrane, Macular hole, Macular peel, Micro-incision vitrectomy surgery, MIVS

## Abstract

**Background:**

To evaluate visual and safety outcomes for 25-gauge (25G) and 27-gauge (27G) micro-incision vitrectomy platforms (MIVS) for the treatment of epiretinal membrane and full-thickness macular holes.

**Methods:**

Retrospective analysis of all patients who underwent internal limiting membrane (ILM) peel surgery from January 2017 through December 2018. 207 cases met the eligibility criteria for inclusion. Primary endpoint was post-operative Best-Corrected Distance Visual Acuity (BCVA) at 6 months.

**Results:**

For all patients combined, mean logMAR BCVA improved from 0.57 (± 0.40) to 0.37 (± 0.36) post-operatively (p < 0.001). For 25G ERMs, logMAR BCVA improved from 0.51 (± 0.28) to 0.30 (± 0.25) post-operatively (p < 0.001). For 27G ERMs, logMAR BCVA improved from 0.33 (± 0.28) to 0.28 (± 0.27) post- operatively (p = 0.15). For 25G FTMHs, logMAR BCVA improved from 0.87 (± 0.48) to 0.51 (± 0.44) post-operatively (p < 0.001). For 27G FTMHs, logMAR BCVA changed from 0.89 (± 0.47) to 0.96 (± 0.60).

**Conclusion:**

Final visual outcomes improved for both 25G and 27G ERM groups and the 25G FTMH group. Both 25G and 27G were safe and well tolerated MIVS platforms for the treatment of ERM and FTMH.

## Introduction

Micro-Incision Vitrectomy Surgery (MIVS) offers an excellent safety profile for patients, while the newest 27-gauge (27G) systems have potential additional safety and outcome benefits when compared to earlier MIVS platforms. This innovation towards progressively smaller instrumentation is playing a major role in the evolution of posterior segment surgery by allowing for decreased incision size and, possibly, less pain, [1, 2] fewer post-surgical wound leaks and subsequently, faster recovery times [3–5] with a low risk of other post-operative complications (6–8). Specifically, multiple large cohort studies and a 2013 literature review concluded there was no increased risk of endophthalmitis when comparing MIVS to 20-gauge (20G) vitrectomy [9–12]. However, a recent meta-analysis highlighted a potential increased risk of endophthalmitis in 25G vs. 20G. The authors hypothesized early vertical sclerotomy (rather than beveled trocar incisions) and hypotony in post-operative fluid-filled eyes (without gas tamponade) were potential risk factors. [13] Since introduced in 2010 by Oshima et al. [14], the range of vitreoretinal conditions for which 27G instrumentation has been utilized is increasing, with many surgeons using 27G for macular epiretinal membrane (ERM) peels and full thickness macular holes (FTMH), as well as more complex cases such as proliferative vitreoretinopathy and retinal detachment repairs [15, 16].

Several prior studies have compared 27G with other MIVS systems for various indications and they generally suggest that key outcomes between 25-gauge (25G) and 27G are comparable [17–21]. However, it is worth noting that most of these studies have relatively small sample sizes and the majority have been conducted in patient populations in Europe or East Asia. Currently, most vitreoretinal surgeons in the US are still primarily operating with the 25G system. Given the relatively recent integration of 27G into clinical practice in the United States, more large scale studies are required. The purpose of this study is to compare visual and safety outcomes for the 25G vs 27G MIVS platforms in the treatment of ERM and FTMH.

## Methods

This Institutional Review Board (IRB) approved single center retrospective study reviews surgical outcomes following 25G and 27G Pars Plana Vitrectomy (PPV) and Internal Limiting Membrane (ILM) peel surgeries for ERM and FTMH. The Oregon Eye Consultants, LLC database was searched using vitrectomy procedure codes for ERM (67041) and FTMH and ILM peel (both 67042). Charts of 379 patients who underwent surgery from January 2017 through December 2018 were reviewed. 207 cases met the eligibility criteria for inclusion (Table 1).

**Table 1:** Eligibility criteria

Inclusion	Exclusion
Visually significant Epiretinal Membrane or Macular Hole	Ocular trauma in study eye
Pre-operative OCT of the operative eye	History of amblyopia in study eye
Post-operative OCT of the operative eye	History of complicated cataract surgery in study eye
Pseudophakic or phakic patients	History of PPV in study eye
Any surgical technique for ILM removal (forceps, loops, peel technique)	History of vitreous hemorrhage in study eye
ILM stain with Brilliant Blue G or Indocyanine Green	History of ocular diseases (i.e. retinal vascular occlusion, high myopia, glaucoma, neoplastic, or chronic inflammatory disorders)
Both eyes of a patient can be enrolled in study	Patients with systemic diseases (i.e. uncontrolled HTN or DM) with ocular evidence of complications (Diabetic Macular Edema, Non-Proliferative Diabetic Retinopathy, Microaneurysms, Dot Blot Hemorrhages, Exudates, Cotton Wool Spots)
Macular membrane peeling with contact lens with direct visualization	History of cataract surgery in study eye within 3 months to pre-operative assessment
	History of YAG capsulotomy in study eye within 1 month of preoperative assessment

*DM* diabetes mellitus, *HTN* hypertension, *ILM* internal limiting membrane, *OCT* optical coherence tomography, *PPV* pars plana vitrectomy, *YAG* Yttrium–Aluminum Garnet

The primary endpoint was Best-Corrected Distance Visual Acuity (BCVA) after 6 months (mean follow-up = 5.9 months). Secondary endpoints included intraocular pressure (IOP), post-operative complications (including need for repeat surgery), and Optical Coherence Tomography (OCT) for central foveal thickness (CFT) and macular volume (MV). Secondary efficiency measurements included surgical time.

Statistical analysis was performed with Microsoft Excel using t-tests or the Wilcoxon signed rank test when appropriate for dependent data, and a *p*-value of < 0.05 was considered statistically significant. This research adhered to the tenets of the Declaration of Helsinki and a waiver of informed consent was granted by the IRB.

## Results

The study included 207 eyes from 105 females and 96 males (mean age 72.6 ± 8.9; range 24–98 years), Table 2. 88 eyes were phakic and 119 were pseudophakic pre-operatively. Of the 207 surgeries, 150 were for ERM and 57 for FTMH; PAK performed 102 surgeries, JWK 43, RMB 44 and AAH 18, respectively. For the ERM group, 102 surgeries were with 25G and 48 with 27G. For the FTMH group, 47 surgeries were with 25G and 10 with 27G. At baseline, there were no statistically significant differences in age, gender or pre-operative lens status between the 25G and 27G groups for ERM or FTMH. For the ERM cohort, there was a statistically significant difference in pre-operative BCVA between 25 and 27G groups (highlighted, Table 2). There were borderline but non-statistically significant differences between IOP and CFT in the ERM 25G and 27G groups.

**Table 2:**
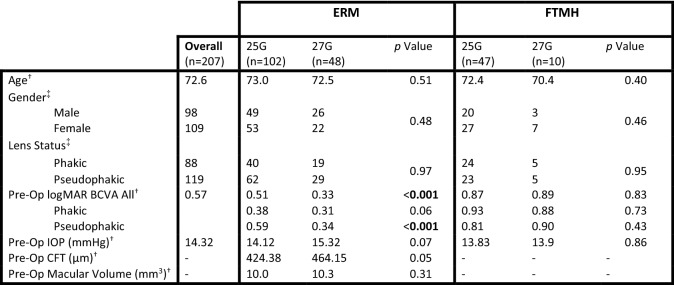
Baseline characteristics and mean pre-operative values

*BCVA* best corrected visual acuity, *CFT* central foveal thickness, *ERM* epiretinal membrane, *FTMH* full thickness macular hole, *IOP* intraocular pressure, *25G* 25-gauge, *27G* 27-gauge

^a^Mann-Whitney U Test

^b^Chi Squared Test

For all patients combined, mean logMAR BCVA improved from 0.57 (± 0.40) to 0.37 (± 0.36) post-operatively (p < 0.001) (Table 3). For 25G ERMs, logMAR BCVA improved from 0.51 (± 0.28) to 0.30 (± 0.25) post-operatively (p < 0.001). For 27G ERMs, logMAR BCVA improved from 0.33 (± 0.28) to 0.28 (± 0.27) post-operatively (p = 0.15). For 25G FTMHs, logMAR BCVA improved from 0.87 (± 0.48) to 0.51 (± 0.44) post-operatively (p < 0.001). For 27G FTMHs, logMAR BCVA changed from 0.89 (± 0.47) to 0.96 (± 0.60).

**Table 3:** Comparison of baseline data and post-operative outcomes (mean values) (statistically significant results highlighted in italics)

	Overall (n = 207)	ERM		FTMH	
		25G (n = 102)	27G (n = 48)	25G (n = 47)	27G (n = 10)
Pre-Op logMAR BCVA All	0.57	0.51	0.33	0.87	0.89
Phakic		0.38	0.31	0.93	0.88
Pseudophakic		0.59	0.34	0.81	0.90
Post-Op logMAR BCVA All	*0.37*	*0.30*	0.28	*0.51*	0.96
Phakic		0.30	0.34	*0.69*	1.00
Pseudophakic		*0.30*	*0.24*	*0.33*	0.91
Pre-Op IOP (mmHg)	14.32	14.12	15.45	13.83	13.9
Post-Op IOP	14.59	14.62	16.42	13.38	16
Pre-Op CFT (µm)	–	424.38	464.15	–	–
Post-Op CFT (µm)	–	*358.58*	*394.37*	–	–
Pre-Op macular volume (mm^3^)	–	10.0	10.3	–	–
Post-Op macular volume (mm^3^)	–	*8.86*	*9.08*	–	–
Surgical time-all data (mins)	27	26	*22.1*	32.5	*36.7*
Surgical time-modified (mins)	27	26	*22.1*	32.5	*29.2*

*BCVA* best corrected visual acuity, *CFT* central foveal thickness, *ERM* epiretinal membrane, *FTMH* full thickness macular hole, *IOP* intraocular pressure, *25G* 25-gauge, *27G* 27-gauge, *Surgical Time-Modified* removed outlier of FTMH 27G data

For 25G ERM phakic patients only, mean logMAR BCVA improved from 0.38 (± 0.2) to 0.30 (± 0.29) (p = 0.09). For 27G ERM phakic patients only, mean logMAR BCVA changed from 0.31 (± 0.3) to 0.34 (± 0.31). For 25G ERM pseudophakic patients, mean logMAR BCVA improved from 0.59 (± 0.3) to 0.30 (± 0.22) (p < 0.001). For 27G ERM pseudophakic patients, mean logMAR BCVA improved from 0.34 (± 0.28) to 0.24 (± 0.25) (p = 0.029).

For 25G FTMH phakic patients only, mean logMAR BCVA improved from 0.93 (± 0.48) to 0.69 (± 0.53) (p = 0.021). For 27G FTMH phakic patients only, mean logMAR BCVA changed from 0.88 (± 0.63) to 1.00 (± 0.59). For 25G FTMH pseudophakic patients, mean logMAR BCVA improved from 0.81 (± 0.48) to 0.33 (± 0.20) (p < 0.001). For 27G FTMH pseudophakic patients, mean logMAR BCVA changed from 0.90 (± 0.3) to 0.91 (± 0.68).

For 25G ERMs, CFT reduced from 424.38 (± 89.24 μm) to 358.58 (± 61.8 μm) (p < 0.001) and MV reduced from 10.0 (± 1.17) mm^3^ to 8.86 (± 0.76) mm^3^ (p < 0.001). For 27G ERMs, CFT reduced from 464.15 (± 75.05) μm to 394.37 (± 42.76) μm (p < 0.001) and MV reduced from 10.3 (± 1.32) mm^3^ to 9.08 (± 0.66) mm^3^ (p < 0.001).

For ERMs, mean surgical time was 3.9 min shorter in the 27G group than in the 25G group (p < 0.001). For FTMH, there was one significantly longer case in the 27G cohort that skewed results disproportionately to the general data distribution for both 27G and 25G. A second analysis was done removing the one case from surgical time, Table 3. Using the data with the one removed surgical time for 27G, mean surgical time was 3.3 min shorter for 27G FTMH than 25G FTMH (p = 0.04). There was no significant difference in IOP in any group post-operatively.

Overall, there were no serious intra-operative complications in any group and no cases of endophthalmitis were documented. Air-fluid exchange was used for incisional tamponade, while gas was used in ERM cases when a retinal tear was identified (Additional file 1: Table S1). There were more retinal tears (4:1) in the 25G:27G ERM group. However, the 25G group is a larger sample size and this study was not designed nor powered to assess statistical significance of intra-operative findings that did not alter primary or secondary outcomes (e.g. post-surgical retinal detachments or recurrence of macular membranes). None of the patients in the ERM group underwent repeat surgeries within 3 months. A total of three patients in the FTMH group required repeat surgery within 3 months. Two of these patients were in the 27G FTMH group, with one requiring surgery for a new FTMH and one requiring surgery for a recurrent non-closing FTMH. The patient in the 25G FTMH also had a recurrent non-closing FTMH. All three FTMH were successfully closed following a period of silicone oil tamponade.

## Discussion

As expected, BCVA significantly improved for all patients combined. For ERM only, the final visual outcomes for 25G vs 27G presented here show that both groups ultimately achieve an almost identical mean BCVA (0.30 vs 0.28) (20/40 vs 20/38). However, for the 27G ERM group these findings were not statistically significant, which is most likely due to the difference in baseline BCVA (0.51 vs 0.33) (20/65 vs 20/43) resulting in a smaller magnitude of improvement. Interestingly, at baseline the 27G ERM group had a borderline statistically significant greater mean CFT, yet counterintuitively a significantly better mean pre-operative BCVA. This appears to be primarily driven by the difference between the 25G and 27G ERM pseudophakic sub-groups at baseline (Table 2). For the 25G vs 27G FTMH, only the 25G group achieved a statistically significant improvement in BCVA.

When stratified by lens status, pseudophakic patients typically achieved better post-operative BCVA outcomes, although a major component of this is likely attributable to the progression of cataract following vitreoretinal surgery in patients who were phakic pre-operatively. A further study may be beneficial to follow these patients for a longer post-operative window in order to capture BCVA outcomes following cataract extraction and IOL insertion after vitreoretinal surgery.

The significantly shorter mean surgical time demonstrated with 27G instrumentation for both ERM and FTMH is encouraging and could be partially attributed to the greater functionality associated with the 27G system versus the 25G system or the surgeon’s greater ease at achieving wound closure after trocar removal with a smaller gauge. However, this does appear to contradict other studies that have generally demonstrated a small increase in surgical time with the 27G system [4, 19, 22].

The strict exclusion criteria chosen for this study may be considered as a limitation, given that 45% of identified cases were excluded. However, the underlying rationale aimed to minimize confounding variables and more accurately elucidate any effect of differing instrumentation gauge on safety and visual outcomes for both ERM and FTMH. Subsequently, the sample sizes for the 27G ERM and FTMH groups were reduced due to the large number of cases excluded. Evidently, the small sample size for the 27G FTMH group in particular poses difficulty in assessing statistical and clinical significance. Thus, more 27G FTMH cases are required to establish whether or not the statistically significant improvement in BCVA seen in the 25G FTMH cases translates to 27G. A recent study of 87 patients with macular holes demonstrated that over a 10 year follow-up period, BCVA continued to improve for 3 years before stabilizing following ILM peel surgery. It also identified that re-establishment of retina ellipsoid zone and the external limiting membrane on post-operative OCT were associated with better outcomes [23]. Longer timepoints in this study would plausibly be beneficial to determine the absolute benefit of these interventions.

At 6-month follow up, both 25G and 27G were safe and well tolerated MIVS platforms for the treatment of ERM and FTMH. Innovation and evolution are constant processes in medical science and technology, which translate to iterative improvements in surgical instrumentation. As such, there is inevitably a learning curve associated with becoming familiar with any new surgical platform and variability in technique between surgeons could also impact outcomes. While this dataset does not show a clear advantage to the newer 27G system, it is interesting to note that all of the retina surgeons in this practice have uniformly switched over to the smaller gauge system for a predominance of their surgical volume. Perhaps, the rationale for the change will become apparent in a prospective study, or a larger sample size with longer follow-up, but those remain to be performed. This study does confirm that for this retrospective series there is no significant difference in outcomes between the two most recent MIVS platforms for ERM and FTMH. Future efforts will be important to better elucidate any plausible significant differences.

## Supplementary information

**Additional file 1: Table S1.** Intra-operative variables.

## Data Availability

Coded dataset available upon request.
