# Supplementary material for: 25 vs. 27-gauge micro-incision vitrectomy surgery for visually significant macular membranes and full-thickness macular holes: a retrospective study
Source: Int J Retina Vitreous. 2020 Nov 16;6:56. doi: 10.1186/s40942-020-00259-4 (PMC7670804; doi:10.1186/s40942-020-00259-4)
Supplement: Supplementary file 1 — Additional file 1: Table S1. Intra-operative variables. [file 40942_2020_259_MOESM1_ESM.docx]

|  | Overall  (n=207) | 25 ERM (n=102) | 27 ERM  (n=48) | 25 FTMH (n=47) | 27 FTMH  (n=10) |
| --- | --- | --- | --- | --- | --- |
| Stain |  |  |  |  |  |
| BBG | 145 | 77 | 48 | 11 | 9 |
| BBG + Kenalog | 62 | 25 | 0 | 36 | 1 |
| Tamponade |  |  |  |  |  |
| None | 73 | 36 | 37 | 0 | 0 |
| AFx | 74 | 62 | 10 | 2 | 0 |
| SF6 | 38 | 3 | 1 | 25 | 9 |
| C3F8 | 22 | 1 | 0 | 20 | 1 |
| Additional Treatments |  |  |  |  |  |
| None | 176 | 92 | 44 | 32 | 8 |
| Endolaser | 11 | 9 | 0 | 2 | 0 |
| Cryotherapy | 19 | 0 | 4 | 13 | 2 |
| Combination | 1 | 1 | 0 | 0 | 0 |

AFx = Air-Fluid Exchange; BBG = Brilliant Blue G; C3F8 = Perfluoropropane; ERM = Epiretinal Membrane; FTMH = Full-Thickness Macular Hole; SF6 = Sulfurhexfluoride

**Additional file 1: Table S1**: Intra-operative Variables
